# Choice of antiretroviral therapy differentially impacts survival of HIV-infected CD4 T cells

**DOI:** 10.1186/2052-8426-2-1

**Published:** 2014-01-03

**Authors:** Nathan W Cummins, Amy M Sainski, Sekar Natesampillai, Gary D Bren, Andrew D Badley

**Affiliations:** Division of Infectious Diseases, Mayo Clinic Rochester, Rochester, MN USA; Department of Molecular Pharmacology and Experimental Therapeutics, Mayo Clinic Rochester, Rochester, MN USA

## Abstract

**Background:**

HIV eradication strategies are now being evaluated *in vitro* and *in vivo*. A cornerstone of such approaches is maximal suppression of viral replication with combination antiretroviral therapy (ART). Since many antiretroviral agents have off target effects, and different classes target different components of the viral life cycle, we questioned whether different classes of ART might differentially affect the survival and persistence of productively HIV-infected CD4 T cells.

**Methods:**

*In vitro* infections of primary CD4 T cells using clinical isolates of HIV-1 that were either protease inhibitor susceptible (HIV PI-S), or resistant (HIV PI-R) were treated with nothing, lopinavir, efavirenz or raltegravir. Cell viability, apoptosis, and the proportion of surviving cells that were P24 positive was assessed by flow cytometry.

**Results:**

In HIV PI-S infected primary cultures, all three antiretroviral agents decreased viral replication, and reduced the total number of cells that were undergoing apoptosis (P < 0.01) similarly. Similarly, in the HIV PI-R infected cultures, both efavirenz and raltegravir reduced viral replication and reduced apoptosis compared to untreated control (P < 0.01), while lopinavir did not, suggesting that HIV replication drives T cell apoptosis, which was confirmed by association by linear regression (P < 0.0001) . However since HIV protease has been suggested to directly induce apoptosis of infected CD4 T cells, and HIV PI are intrinsically antiapoptotic, we evaluated apoptosis in productively infected (HIV P24+) cells. More HIV p24 positive cells were apoptotic in the Efavirenz or raltegravir treated cultures than the lopinavir treated cultures (P = 0.0008 for HIV PI-R and P = 0.06 for the HIV PI-S), indicating that drug class impacts survival of productively infected CD4 T cells.

**Conclusions:**

Inhibiting HIV replication with a PI, NNRTI or INSTI reduces total HIV-induced T cell apoptosis. However, blocking HIV replication with PI but not with NNRTI or INSTI promotes survival of productively HIV-infected cells. Thus, selection of antiretroviral agents may impact the success of HIV eradication strategies.

**Electronic supplementary material:**

The online version of this article (doi:10.1186/2052-8426-2-1) contains supplementary material, which is available to authorized users.

## Background

The major barrier to HIV eradication is latent virus contained within resting cells. Because these cells contain integrated HIV provirus and do not replicate virus, they are resistant to agents which inhibit viral replication, and they are not affected by immune effector mechanisms that target viral proteins. One approach to eradicating these cells involves reactivating HIV [1, 2], which to be of benefit, must cause the death of cells harboring HIV. In a recent report using a primary cell model of latent infection with a green fluorescent protein–labeled NL4.3ΔNefΔPol virus cells with SAHA-mediated reactivation of HIV did not die during 18 days of observation, even when co-incubated with autologous CD8 T cells [3]. Another study evaluated latently infected central memory CD4 T cells (TCM) cells; reactivation with interleukin-2 and interleukin-7 did not cause cell death, whereas reactivation with CD3-CD28 co-stimulation did kill cells [4], indicating that it is possible to reactivate HIV from latency in a manner which causes the death of latently infected cells. Thus, as strategies are being developed to test this in patients, it will be critical to know if exogenous factors modify the ability of these cells to be killed.

The so-called “shock and kill” [5] theoretical approach to HIV eradication invariably involves co-administration of antiretroviral medications to prevent further rounds of infection and repopulation of the latent reservoir. The latent reservoir consists principally of integrated provirus within central memory CD4 T cells. These are thought to arise in one of two non-mutually exclusive ways: direct infection of central memory cells, or infection of activated cells which then revert to a memory phenotype. These models predict that the size of the latent reservoir is proportional to the cumulative number of productively infected cells. This prediction has been tested and verified *in vivo*[6–8]. Therefore, it is of interest to understand factors which influence the size of this ‘active reservoir’ of HIV.

A variety of reports suggest that different classes of antiretroviral agents have off target effects [9, 10] and differ in their ability to impact apoptosis – for example efavirenz has been suggested to be pro-apoptotic [11], whereas peptidomimetic protease inhibitors (eg. lopinavir) have been suggested to be anti-apoptotic [12]. Indeed the anti-apoptotic effects of PI have been used to reverse non-HIV disease processes characterized by excessive apoptosis [13–15]. Moreover, since HIV protease can directly induce HIV infected-cells to die [16–18], we hypothesized that protease inhibitors might inhibit HIV infected cell death. Therefore, we tested different representative antiretrovirals’ abilities to impact the survival of HIV infected cell cultures, or specifically productively HIV infected cells, and assessed whether these effects were dependent upon inhibition of viral replication.

## Methods

### Cell culture and infection

Jurkat and primary CD4 T cells (American Type Culture Collection, Manassas, VA) were cultured in RPMI 1640 (Mediatech Inc, Manassas, VA) supplemented with 10% fetal calf serum (Atlanta Biologics, Atlanta, GA) and 2 mM L-glutamine at 37°C and 5% CO2. Peripheral blood mononuclear cells were obtained from leukoreduction system chambers from anonymous, HIV-negative apheresis donors via an Institutional Review Board (Mayo Clinic Institutional Review Board) approved protocol [19]. Primary CD4 T cells were isolated using RosetteSep® Human CD4+ T Cell Enrichment Cocktail (Stemcell Technologies, Vancouver BC, Canada) per manufacturer’s protocol, and cultured with 1 μg/ml phytohemagglutinin (Remel, Lexena, KS) and 50 IU/ml interleukin-2 (Chiron Corporation, Emeryville, CA) for 24 hours. HIV-1 strains AD.MDR01 (PI-resistant), and Donor E (PI-susceptible) (NIH AIDS Reagent Program) were used to infect primary CD4 T cells as previously described [20]. The genotype of the PI-resistant strain has been previously published [21]. Immediately following infection, cells were treated with or without – efavirenz (10 nM), lopinavir (100 nM), or raltegravir (100 nM) (NIH AIDS Reagent Program).

### Cell free procaspase 8 cleavage assay

To assess the effect of the various antiretrovirals on the proteolytic activity of HIV protease, a fluorometric assay was used for the cleavage of a consensus site on procaspase 8 peptide, performed as previously described [18]. The fluorogenic peptide for the cleavage site at Caspase8 to yield Casp8p41 was Arg-Glu(EDANS)-Pro-Lys-Val-Phe-Phe-Ile-Gln-Ala-Lys(DABCYL)-Arg.

### P24 measurement

HIV-1 p24 antigen ELISA (ZeptoMetrix, Buffalo, NY) was performed on cell culture supernatants according to manufacturer’s protocol and read on an EL800 plate reader (BioTek, Winooski, VT).

### Flow cytometry

Apoptosis was measured by Terminal deoxynucleotidyltransferase-mediated dUTP-biotin nick end labeling (TUNEL) (Roche, St. Louis, MO), according to the manufacturer’s protocol. Viability was determined cells with LIVE⁄DEAD® Fixable Dead Cell Stain Kit (Molecular Probes, Life Technologies, Grand Island, NY) according to the manufacturer’s protocol. Cells were fixed with 2% paraformaldehyde, permeabilized with 0.01% NP-40 in phosphate buffered saline (PBS), and stained with a PE-conjugated anti-HIV p24 monoclonal antibody (Clone KC57, Beckman Coulter, Mervue Galway, Ireland) at a 1:1000 dilution in 5% bovine serum albumin in PBS. Isotype antibodies were used for negative controls. Flow cytometry was performed on a FACScan or LSRii flow cytometer (Becton Dickinson, Franklin Lakes, NJ), and data analyzed with FlowJo software (Tree Star, Inc, Ashland, OR).

### Statistical analysis

Data are expressed as means +/- standard error. Values were compared across samples by t-test or one way ANOVA as appropriate. Correlations were performed by linear regression analysis. A p-value < 0.05 was considered statistically significant. Statistical analyses were performed using GraphPad InStat software (Graphpad Software Inc, La Jolla, CA).

## Results

One of the earliest observations about the immunopathology of HIV was that HIV infection results in the apoptotic death of both HIV infected and HIV uninfected (bystander) cells [22]. Reducing HIV replication by any means reduces this effect, however there are at least two theoretical reasons by which protease inhibitors (eg lopinavir) might reduce apoptosis to a greater extent than an NNRTI (eg efavirenz) or an integrase inhibitor (eg raltegravir) – 1) through stabilization of the mitochondrial outer membrane pore, thereby blocking all mitochondrial-dependent forms of apoptosis, as has been experimentally shown [23]) and 2 theoretically through inhibition of HIV protease mediated cleavage of procaspase 8, which generates proapoptotic Casp8p41 [16, 17, 24]. To assess whether lopinavir indeed inhibits generation of Casp8p41, we used a validated *in vitro* assay which measures the cleavage of a procaspase 8 octapeptide representing the cleavage site within procaspase 8 which is cleaved to generate Casp8p41 [18]. Lopinavir (100 nM) decreased HIV protease cleavage of procaspase 8 by >90% compared to untreated control, whereas a lower dose (10 nM) of lopinavir, raltegravir (10 and 100 nM), and efavirenz (1 and 10 nM) did not (Additional file 1: Figure S1). Since Casp8p41 is only present in productively-infected cells [17], these data suggest that lopinavir may inhibit apoptosis of productively-infected cells through this unique mechanism.

**Figure 1:**
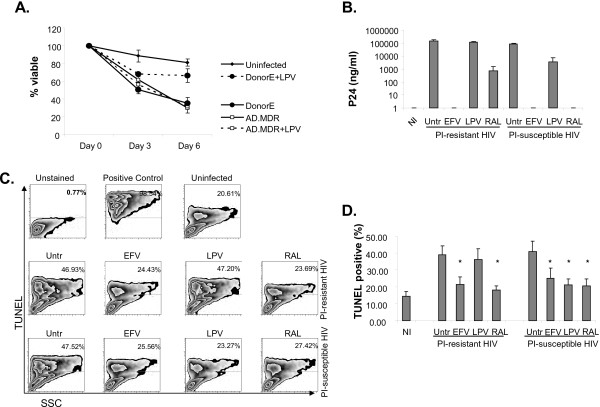
**Primary CD4 T cells were** ***in vitro*** **infected with the PI-resistant AD.MDR01 and the PI-susceptible Donor E HIV-1 strains in the presence or absence of efavirenz, lopinavir or raltegravir. A)** Cell viability over time as measured by trypan blue exclusion. **B)** HIV p24 concentration in cell culture supernatants was measured by ELISA at day 6 post infection. **C)** Apoptosis as measured by TUNEL staining and flow cytometry at day 6 post infection; representative results of three independent experiments. **D)** The percent of cells TUNEL positive at day 6 post infection, expressed as mean +/- standard error of three independent experiments, was compared by ANOVA. * indicates a significant P value <0.05 compared to untreated infected cells.

Next we assessed whether raltegravir, efavirenz, and lopinavir would have different effects on apoptosis associated with HIV infection, or apoptosis in HIV-infected cells. Primary CD4 T cells from HIV-uninfected donors were infected *in vitro* with two clinical isolates of HIV-1 – a PI-resistant (HIV PI-R) strain and a PI-susceptible (HIV PI-S) strain – in the presence or absence of single antiviral agents. In the absence of antiviral agents, infection of primary CD4 T cells with the PI-S and the PI-R strain resulted in a similar loss of viability over time (35.5 ± 6.3% and 30.2 ± 5.9% viable at day 6 respectively, vs 81.1 ± 4.1% viable in the mock infected, P < 0.001, Figure 1A), and similar viral replication, as assessed by p24 antigen production at day 6 (83469 ± 16801 pg/ml and 147522 ± 28382 pg/ml, P = 0.07, Figure 1B). These data suggested that the PI-S and PI-R viruses had similar fitness and pathogenicity in our model.

Treatment of the HIV PI-S infected cultures with lopinavir, raltegravir, and efavirenz resulted in 95.5%, 100% and 100% reduction in p24 production by day 6 (Figure 1B). However, as expected, treatment of the HIV PI-R cultures with lopinavir did not reduce viral replication (122708 ± 8404 pg/ml of p24), whereas treatment with raltegravir and efavirenz resulted in 99.5% and 100% reduction in p24 production by day 6 compared to control treated infected cells (Figure 1B). These data confirmed that the HIV PI-R virus was in fact resistant to the antiviral activity of lopinavir in our model.

**Figure 2:**
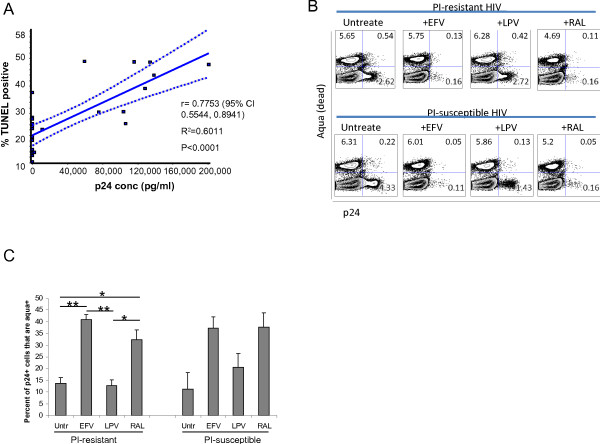
**Lopinavir specifically promotes survival of productively HIV-infected CD4 T cells. A)** The percent of TUNEL positive cells at day 6 was regressed upon supernatant p24 concentration using linear regression. P < 0.05 considered statistically significant. **B)** Intracellular HIV p24 expression as a marker of cell infection and cell death (Aqua stain positive) were measured by flow cytometry. Representative results at day 2 post infection of 3 independent experiments. **C)** The percentage of p24+ cells that co-stained positively for the Aqua dead stain, expressed as the mean +/- standard error for three independent experiments, was compared using ANOVA. *Indicates a p value <0.05, **a p value <0.01 in post-tests between groups.

Both the HIV PI-S and the HIV PI-R infections resulted in more apoptosis, as measured by TUNEL positivity, compared to mock infected cells at day 6 (40.7 ± 6.1% and 38.9 ± 5.2%, vs 14.5 ± 2.7% in the mock infected, P < 0.001, Figure 1C and D). In the HIV PI-S infected cultures, treatment with lopinavir, raltegravir, and efavirenz reduced TUNEL positivity compared to untreated, infected cells (21.2 ± 3.5%, 20.7 ± 4.1, and 24.9 ± 6.1% TUNEL positive respectively, P < 0.01 compared to 40.8 ± 6.2% in HIV alone). There was no difference between treatments (P > 0.05), and the degree of apoptosis was not different than mock infected cells (P > 0.05). However, in the HIV PI-R infected cultures, treatment with lopinavir did not reduce TUNEL positivity (36.1 ± 6.5%, P > 0.05 compared to 38.9 ± 5.2% in HIV alone), whereas raltegravir and efavirenz treatment did reduce TUNEL positivity to levels similar to uninfected cells (18.2 ± 2.5% and 21.3 ± 4.4%, P < 0.01 compared to untreated infected cells). These data suggest that blocking viral replication by agents of any class is sufficient to reduce global HIV-associated apoptosis. In fact, TUNEL positivity at day 6 post infection was strongly and significantly correlated with p24 production across samples (r = 0.7753, P < 0.0001, Figure 2A), regardless of infecting strain or class of antiretroviral.

We next questioned whether similar results would be seen specifically in productively-infected cells, which constitute the major reservoir of ongoing viral replication even on “suppressive” cART [25]. HIV-infected cell death and intracellular expression of HIV p24 was reduced in the HIV PI-R infected cultures by efavirenz or raltegravir, but not lopinavir. In the cultures infected with HIV PI-S reductions in intracellular p24 expression were seen with all three antivirals (Figure 2B). Interestingly, at 48 hours post infection, treatment with efavirenz or raltegravir resulted in a two- to three-fold increase in the proportion of p24 positive cells that were dead, compared to untreated or lopinavir treated p24 positive cells (P = 0.0008 for HIV PI-R and P = 0.06 for the HIV PI-S by ANOVA, Figure 2B and C). Similar results were not seen at later time points through day 6 post infection (data not shown). These data are consistent with PI-mediated inhibition of apoptosis [15], and NNRTI-induction of apoptosis [11] in productively-infected cells and Pr inducing apoptosis in infected cells.

## Discussion

It is widely accepted that total CD4 T cell apoptosis following *in vitro* HIV infection is proportional with viral replication, and therefore it is not surprising that inhibiting HIV replication with lopinavir, efavirenz or raltegravir results in similar reductions in total HIV-associated apoptosis. However, in the current report we demonstrate that important differences exist between these drugs in terms of their impact on apoptosis of productively HIV-infected cells; HIV PI promote the survival and persistence of productively infected cells. The *in vivo* correlate of this finding is untested and unknown, but potentially the clinical use of a PI might increase the cumulative burden of cells containing HIV, when compared to non-PI containing regimens. If true this could greatly impact the likelihood of eradicating HIV in different patient populations, and therefore should be directly assessed in large, carefully controlled clinical studies.

Combination ART results in decreased apoptosis of CD4 T cells that correlates with a reduction in viral load and reductions in immune activation in peripheral blood [26, 27] and lymph nodes [28]. However, the relative contribution of individual components of regimens remains unclear. The NRTIs azidothymidine, didanosine, lamivudine and stavudine are to varying degrees pro-apoptotic *in vitro* and *in vivo*[29–32]. Similarly, treatment of T cells *in vitro* with supratherapeutic concentrations of efavirenz results in increased apoptosis compared to untreated cells [11]. On the other hand, PIs decrease apoptosis in HIV infected CD4 T cells *in vitro* and *ex vivo* through inhibition of mitochondrial transmembrane potential loss [23], independently of the antiviral effect [12]. PIs also decrease apoptosis in animal models of non-HIV related diseases associated with excessive apoptosis, such as sepsis [13], Fas-induced hepatitis, stroke [14], and retinal degeneration [15]. This differential effect on apoptosis was recently demonstrated clinically in virologically suppressed HIV-infected patients on a PI-containing regimen who had a decrease in intrinsic apoptosis of peripheral blood mononuclear cells over time, whereas patients on an NNRTI-regimen did not [33]. The effects of entry inhibitors (maraviroc), fusion inhibitors (enfuvirtide), and integrase inhibitors, on apoptosis in HIV-infected cells have not been extensively studied previously.

Our data complement the recent advances in our understanding of death occurring in abortively HIV-infected, non-permissive CD4 T cells [34]. They demonstrate that entry inhibitors and efavirenz permit survival of non-permissive, resting CD4 T cells in the face of HIV exposure, whereas AZT and raltegravir permit death of these cells through accumulation of toxic DNA intermediates from incomplete reverse transcription and integration. While this provides a suitable model for a mechanism of “bystander” cell death in untreated HIV-infection, we focused on cell death in permissive (activated), productively infected (ie. p24 positive) cells, as eradication strategies that involve viral reactivation necessarily involve post-integration events in the viral life cycle.

Since our observations were made *in vitro* using only single drug treatments, future studies in patient cohorts on triple drug regimens will be required both to validate our results and to assess these effects in the context of multiple pro and anti -apoptotic mechanisms involved in *in vivo* HIV-infection [35]. Also, since representative medications from three drug classes were studied; additional studies are necessary to establish class effects versus individual drug effects. This is important as we have previously demonstrated that some anti-apoptotic effects of PIs are restricted to peptidomimetic PIs (such as lopinavir, saquinavir and nelfinavir) and may not extend to non-peptidomimetic PIs (such as atazanavir and darunavir) [12]. However, it is likely that the effect demonstrated by lopinavir in this study would be similar with other peptidomimetic PIs. Additional studies are also needed examining various combinations of ART agents and classes.

In the current era where curing HIV infection is being considered, it is now important to reassess the drugs which we use to treat HIV infection to determine how they could impact the size of the HIV reservoir. Inhibiting apoptosis of HIV-infected cells may preserve or increase the size of the latent viral reservoir, whereas increasing apoptosis of HIV-infected cells would predictably decrease the reservoir. Indeed consistent with our observations, cART intensification by the addition of reverse transciptase inhibitors [36], maraviroc [37] or raltegravir [38, 39] to an already suppressive regimen results in modest decreases in the magnitude of the latent reservoir.

## Conclusion

As eradication strategies are being designed, investigators need to be cognizant of the potentially confounding effects of choice of drug class on survival of productively infected cells, particularly as it relates to regulation of apoptosis.

## Electronic supplementary material

Additional file 1: Figure S1: A fluorogenic peptide corresponding to the protease cleavage site in procaspase8 in acetate buffer (pH4.7) with or without indicated concentration of raltegravir (10 nM or 100 nM) or lopinavir (10 nM or 100 nM) or efavirenz (1 nM or 10 nM) were preincubated at 37C for 30 min. HIV-1 protease was added, and cleavage monitored over time by measuring fluorescence. (PPT 212 KB)

## Abbreviations

AIDS: Acquired immunodeficiency syndrome
ANOVA: Analysis of variance
cART: Combination antiretroviral therapy
ELISA: Enzyme linked immunosorbent assay
HIV: Human immunodeficiency virus
INSTI: Integrase strand transfer inhibitor
IU: International Unit
NIH: National Institutes of Health
NRTI: Nucleoside reverse transcriptase inhibitor
NNRTI: Non-nucleoside reverse transcriptase inhibitor
PBS: Phosphase buffered saline
PE: Phycoerythrin
PI: Protease inhibitor
PI-R: Protease inhibitor resistant
PI-S: Protease inhibitor susceptible
SAHA: Suberoylanilide hydroxamic acid
TCM: Central memory T cells
TUNEL: Terminal deoxynucleotidyltransferase-mediated dUTP-biotin nick end labeling.
