# Supplementary figures and images for: Choice of antiretroviral therapy differentially impacts survival of HIV-infected CD4 T cells
Source: Mol Cell Ther. 2014 Jan 3;2:1. doi: 10.1186/2052-8426-2-1 (PMC4448955; doi:10.1186/2052-8426-2-1)

## Slide 1
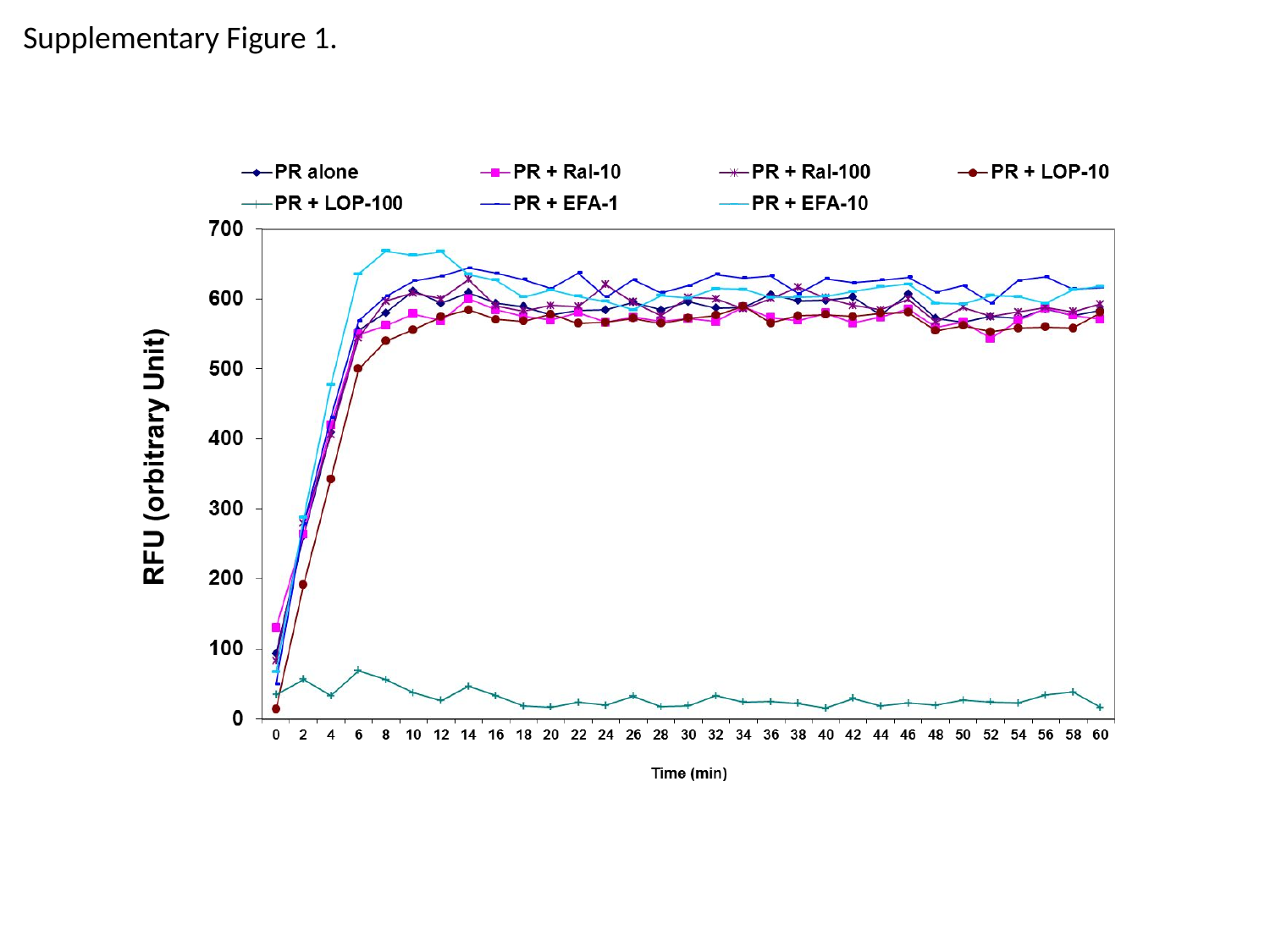

Supplementary Figure 1.

Supplement: Supplementary file 1 — Additional file 1: Figure S1: A fluorogenic peptide corresponding to the protease cleavage site in procaspase8 in acetate buffer (pH4.7) with or without indicated concentration of raltegravir (10 nM or 100 nM) or lopinavir (10 nM or 100 nM) or efavirenz (1 nM or 10 nM) were preincubated at 37C for 30 min. HIV-1 protease was added, and cleavage monitored over time by measuring fluorescence. (PPT 212 KB) [file 40591_2013_5_MOESM1_ESM.ppt]
